# Simultaneous appearance of cerebral venous thrombosis and subdural hematomas as rare cause of headache in puerperium following epidural analgesia: a case report

**DOI:** 10.3325/cmj.2012.53.379

**Published:** 2012-08

**Authors:** Željko Župan, Vlatka Sotošek Tokmadžić, Marinka Matanić-Manestar, Alan Šustić, Igor Antončić, Siniša Dunatov, Ivan Pavlović, Ronald Antulov

**Affiliations:** 1 Department of Anesthesiology, Reanimatology and Intensive care, Medical Faculty, University of Rijeka, Rijeka, Croatia; 2 Department of Neurology, Medical Faculty, University of Rijeka, Rijeka, Croatia; 3 Department of Radiology, Medical Faculty, University of Rijeka, Rijeka, Croatia

## Abstract

The aim of this study is to report the first case of simultaneous appearance of cerebral venous thrombosis (CVT) and bilateral subdural hematomas (SDHs) following epidural analgesia for labor and delivery and to point out the difficulty of establishing such a diagnosis in the presence of postpartum headache. A 26-year old primigravida with a history of epilepsy received epidural analgesia for delivery. Three days after the uneventful spontaneous vaginal delivery she complained about the headache. Patient responded very well to the pain medication and oral hydration, and the headache was relieved. Ten days after the delivery, the headache reoccurred, and an epidural blood patch was performed that successfully relieved her symptom. Stronger progressive headache with nausea reappeared two days later and the parturient was readmitted to hospital. Urgent neuroimaging examinations detected CVT of right the transverse sinus, ipsilateral cortical veins, and partially occluded superior sagittal sinus, as well as bilateral subacute/chronic SDHs. The treatment of the patient with low molecular weight heparin and antiaggregation therapy was effective. In this case, the diagnosis was delayed because of atypical clinical presentation and potentially confounding events (epidural analgesia and assumption that it was a case of PDPH). It is important to carefully observe patients in such conditions and promptly conduct suitable diagnostic tests. Otherwise, unrecognized intracranial complications and delay of appropriate therapy could be life-threatening.

The use of epidural analgesia for pain relief during labor and delivery has become increasingly popular. Although it is considered effective and safe, epidural analgesia could be associated with a wide spectrum of neurological complications. The most common neurological complications include accidental dural puncture and post-dural puncture headache (PDPH), central nervous system infections such as meningitis, occurrence of cerebral venous thrombosis (CVT), or epidural spinal hematoma or intracranial subdural hematomas (SDHs) that can have similar or even identical symptoms such as headache (1-4). Fortunately, neurological complications following epidural analgesia are rare, but if they occur the consequences could be serious (5,6).

CVT and other intracranial events related to the peripartum period pathophysiology may present with a wide spectrum of different neurological symptoms including headache (7,8). The diagnosis of such complications can be delayed and challenging, especially in cases with atypical clinical presentation and concomitant use of regional anesthesia that can lead to misdiagnosis of headache assuming it to be the case of PDPH. We described a 26-year-old parturient who received epidural analgesia for labor and delivery and who simultaneously developed multiple CVT and bilateral SDHs.

## Case report

**Figure 1:**
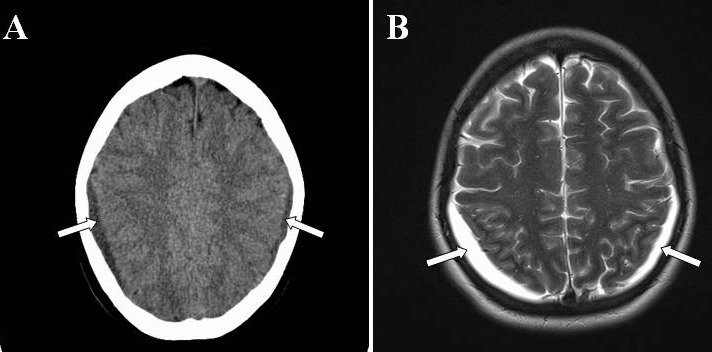
A non-contrast enhanced CT scan (**A**) and axial T2 weighted spin echo magnetic resonance imaging scan (**B**) showing bilateral subdural hematomas (marked by white arrows).

**Figure 2:**
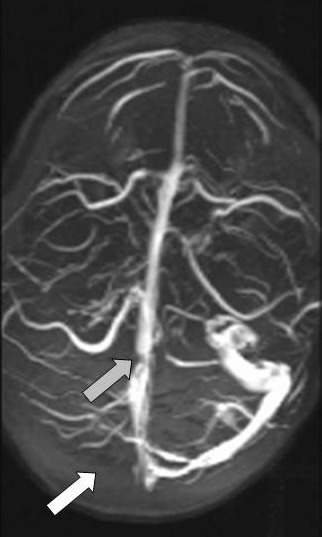
Brain magnetic resonance venography (MRV) showing no flow in the right transverse sinus and ipsilateral cortical veins (white arrow) and partially occluded flow in the superior sagittal sinus (gray arrow).

A 26-year-old primigravida received epidural analgesia for successful vaginal delivery. She had a history of epilepsy since childhood, currently symptom-free. Two days following noncomplicated delivery, she and her healthy child were discharged from the hospital. On the third postpartum day, the patient started to complain about headache that was considered as PDPH, although no evident sign of dural puncture during epidural procedures was observed. She responded very well to recommended bed rest, pain medication (diclofenac, Voltaren, Pliva, Zagreb, Croatia) in a dose of 50 mg twice daily), and additional oral hydration at home, and headache was resolved. One week later, the headache reoccurred and the patient was readmitted to the hospital. The neurological examination was unremarkable and the inspection of epidural puncture site did not show any signs of infections. Blood tests and other biochemical laboratory data (red and white blood counts, metabolic profile, serum electrolytes, blood glucose, blood urea nitrogen, creatinine, arterial blood gas analyses, C-reactive protein) were within the reference range. Persistent PDPH was considered once again although the epidural block was uneventful. Epidural blood patch was performed at the level L3-L4 by using 20 mL of autologous blood. Within an hour, the headache was almost resolved, and the patient was discharged from the hospital on the same day. Two days later, she complained about strong fronto-occipital headache that was worsened in the standing position. The headache was followed by nausea and the patient was immediately readmitted to the emergency department. She was hemodynamic and respiratory stable, arterial blood pressure was normal, biochemical (red and white blood counts with differential tests, erythrocyte sedimentation rate, blood levels of sodium, potassium, chloride, magnesium, calcium, bicarbonate, urea, creatinine, glucose, pH and bicarbonate, arterial carbon dioxide and oxygen concentrations, as well as levels of the serum creatine kinase, C-reactive protein, lactate dehydrogenase, hepatic enzymes, and analysis of the protein electrophoresis) and coagulations (platelets count, prothrombin time, activated partial thromboplastin time and ratio, fibrinogen and antithrombin III levels) tests were within the reference range. She still had no other neurological symptoms and signs except headache. An urgent computerized tomography (CT, Sensation 16, Siemens, Forchheim, Germany) of the brain was performed and revealed bilateral parietal-occipital subacute/chronic SDHs (Figure 1A). The magnetic resonance imaging (MRI, Avanto 1,5 T, Siemens) confirmed the mentioned diagnosis (Figure 1B), and magnetic resonance venography (MRV, Avanto 1,5 T, Siemens) revealed right transverse sinus and right parietal cortical venous thrombosis, as well as partial thrombosis of the superior sagittal sinus (Figure 2).

The patient was transferred to the neurological intensive care unit where she was treated with low molecular heparin (LMWH), enoxaparin (Clexan, Sanofi Aventis, Paris, France), 1 mg/kg subcutaneously every 12 hours during 10 days. After LMWH therapy, the treatment was continued with acetylsalicylic acid (Aspirin, Bayer, Leverkusen, Germany) in a dose of 100 mg daily, and with 75 mg of the dipiridamol (Persantine, Boehringer, Ingelheim, Germany) once a day, during next six months. The dosage of sodium valporate was elevated to 500 mg daily during the hospital stay. Workup for vasculitis, lupus, antiphospholipid syndrome, and specific coagulation disorders was negative. Namely, antinuclear antibody, anti-neutrophil cytoplasmic antibody, anti-dsDNA, and extractable nuclear antigen tests were negative, lupus anticoagulant and other thrombophilic factors were also negative, and the levels of the complements C3 and C4 as well as proteins C and S were within the reference range. During further clinical course, patient remained asymptomatic and after four weeks she completely recovered and was discharged from hospital. A control MRI and MRV that were performed 14 days after hospital readmission showed partial regression of the SDHs with persistent CVT. A control neuroimaging that was made one month after hospital discharge revealed a completely spontaneous resolution of bilateral SDHs, and partial recanalization of the parietal cortical veins, superior sagittal sinus, and right transverse sinus. Three months after hospital discharge MRV examination showed full vein recanalization.

## Discussion

Postpartum period could be a high-risk period for the development of different types of headaches. The overall incidence of postpartum headaches cannot be exactly determined due to limited and inconsistent studies (8-12). However, several studies reported that up to 75% of postpartum headaches are primary (migraines or tension-type headaches) in nature (10,12). Secondary headaches may occur due to intracranial pathologies including CVT, reversible cerebral vasoconstriction syndrome, stroke, intracranial hematomas, meningitis, or cerebral tumors (8). PDPH is the most common complication of obstetric regional anesthesia and the most probable cause of headache in pregnant women if epidural and/or spinal anesthesia or analgesia was applied during delivery.

This case report presents difficulties in diagnosing the cause of postpartum headache in a patient who underwent regional analgesia. The first suspected diagnosis was PDPH although the placement of lumbar epidural catheter and analgesia for labor was uneventful with no obvious sign of dural puncture. Therefore, we started with conventional treatment of PDPH by pain medication, hydration, and a subsequent blood patch insertion. The decision to perform the blood patch was based on the clinical finding that the patient had only headache and no additional neurological symptoms and signs, and that headache still had a postural component. A further increase in the headache severity with progressive nausea after epidural blood patch indicated that this was a case of more serious intracranial pathology, and the patient received diagnostic CT, MRI, and MRV.

To the best of our knowledge, this is the first report of a simultaneous occurrence of CVT and bilateral SDHs in postpartum period following epidural analgesia. Our opinion is that the patient’s headache was a symptom of CVT and SDH due to peripartum pathophysiology, possibly coincidentally observed with regional analgesia, and that the patient unnecessarily received an epidural blood patch. This suggestion is in agreement with the case report by Takahashi et al (13), who described severe persistent headache as a single symptom of CVT followed by acute SDHs in a previously healthy male patient without spinal/epidural anesthesia or analgesia. In our patient, the systemic diseases, as well as specific coagulation disorders were excluded by using immunological, serological, and specific laboratory tests. Possible triggering factors for the appearance of CVT or SDHs in our case could be the significant changes in intracranial pressure, dehydratation, venous congestion, and endothelial damage during delivery in combination with increased hypercoagulability after labor (7). A typical presentation of symptoms related to CVT or SDHs include progressive positional headache often accompanied with other neurological signs and symptoms, such as expressive dysphasia, mental disturbance, poor coordination, weakness of the limbs, loss or disturbance of focal sensation, impairment or loss of speech, consciousness disturbance, clinical manifestations of cerebral herniation, and deep coma with cardiac arrest in the worst case (6,9).

In our patient, the diagnosis was delayed because of atypical presentation of symptoms and because it was assumed that it was a case of PDPH after epidural analgesia. It is evident that headache as a symptom of serious intracranial events in postpartum period can mimic PDPH. Therefore, the rare and potentially fatal cranial peripartum complications can easily be misdiagnosed, particularly in parturient with regional analgesia. As a rule these diagnoses were made after the placement of an epidural blood patch and subsequent persistent and deteriorated headache.

However, the occurrence of CVT and SDHs associated with regional analgesia cannot be excluded. Namely, there are several reports on postpartum CVT (4,14-21) or SDHs (3,6,12,22-30) due to dural puncture after obstetric regional analgesia (Table 1).

**Table 1:** Cerebral venous thrombosis and subdural hematomas in postpartum period following epidural or spinal analgesia and anesthesia: clinical presentations, treatment, and outcome

**References**	**Procedures**	**Clinical presentations**	**Complications**	**Treatment**	**Outcome**
Vaughan DJA et al, 2000 (3)	Epidural analgesia	Occipital headache, tonic-clonic seizures	Subdural hematoma	Conservative	Recovered
Kapessidou Y et al, 2006 (4)	Spinal anesthesia	Frontal headache, dizziness, acute left hemiparesis, blurred vision, somnolence	Thrombosis of the posterior sagittal venous sinus	Conservative	Recovered
Aziz F, 2010 (6)	Epidural analgesia	Headache, cardiac arrest	Subdural hematoma	Resuscitation, conservative	Died
Mashour GA et al, 2006 (12)	Epidural analgesia	Headache, syncopal episode, blurry vision, seizure	Subdural hematomas	Surgery	Recovered
Ravindran RS et al, 1989 (14)	Epidural analgesia	Frontal headache, nausea, vomiting, seizures	Dural sinus thrombosis	Conservative	Recovered
Wittmann M et al, 2012 (21)	Epidural analgesia	Headache	Sinous venous thrombosis	Conservative	Recovered
Stocks GM et al, 2000 (15)	Epidural analgesia	Headache, confusion, sedation	Sinous venous thrombosis	Conservative	Recovered
Zeidan A et al, 2010 (30)	Spinal anesthesia	Headache, associated, right eye tearing, fifth cranial nerve palsy, left hemiparesis	Subdural hematoma	Conservative	Recovered
Kardash K et al, 2002 (25)	Epidural analgesia	Headache, seizures	Subdural hematoma	Conservative	Recovered
Moradi M et al, 2012 (28)	Spinal anesthesia	Headache, nausea, vomiting	Subdural hematoma	Surgical	Recovered
Ezri T et al, 2002 (24)	Epidural analgesia	Headache	Subdural hematoma	Conservative	Recovered
Verdu MT et al, 2007 (26)	Spinal anesthesia	Headache, dysphasia, numbness in upper right limb and face	Subdural hematoma	Conservative	Recovered
Todorov L et al, 2005 (18)	Epidural anesthesia	Headache, seizures	Sinus venous thrombosis	Conservative	Recovered
Davies JM et al, 2001 (23)	Epidural analgesia	Headache, disphasia, deterioration, right-handed dysdiadokinesis	Subdural hematoma	Surgical	Recovered
Diemunsch P et al, 1998 (22)	Epidural analgesia	Headache, focal neurological signs	Bilateral subdural hematomas	Conservative	Recovered
Ghatge S et al, 2008 (19)	Epidural analgesia	Headache, confusion, disphagia, hemiparesis, nystagmus	Superior sagitalis sinus, galen vein and straight sinus thrombosis	Conservative	Recovered
Karci A et al, 2005 (17)	Spinal-epidural anesthesia	Headache, hemiparesis, Babinski sign positive	Superior sagital sinus thrombosis	Conservative	Recovered
Kueper M et al, 2008 (20)	Epidural analgesia	Headache, hemiparesis, hemihypesthesia	Sinus venous thrombosis	Conservative	Recovered
Kulandayan S, 2002 (16)	Epidural analgesia	Headache, hemiparesis, seizures, loss of consciousness 3-5 min	Sinus venous thrombosis	Conservative	Recovered
Liang MY and Pagel PS, 2012 (29)	Epidural analgesia	Headache, radiating pain in legs, paresthesias on the left side of body	Bilateral interhemispheric subdural hematoma	Conservative	Recovered
Dawley B and Hendrix A, 2009 (27)	Spinal anesthesia	Headache, nausea, vomiting	Subdural hematoma	Surgery	Recovered

In our case, since accidental dural puncture was not observed, its presence could not have been safely excluded. The incidence of PDPH after unrecognized dural puncture following epidural analgesia during labor and delivery is very rare and amounts to 0.6% or less (31). Therefore, in such patients at low risk for PDPH, in the case of postpartum headache, other causes of headaches have to be carefully considered, and suitable diagnostic tests have to be performed without delay.

CVT and SDHs are serious events that should be immediately detected and appropriately treated. Treatment with heparin is the first choice upon confirmation of CVT, even in the presence of small intracerebral hemorrhage or small SDHs (32,33). In the reported case, the low molecular weight heparin and antiaggregation treatment were shown as a good therapy choice leading to a successful outcome.

In conclusion, we described a parturient with progressive headache due to simultaneous appearance of CVT and SDHs, as complications related to peripartum pathophisiology, possibly coincidentally observed with epidural analgesia. Our case points out that special attention should be given to differential diagnosis of headaches following epidural analgesia in the purperium, and that neurological examination should be carried out before performing an epidural blood patch, especially when the presentation of symptoms is atypical. Our experience suggests that rapid diagnostic, neuroimaging tests (CT, MRI, and MRV) should be carried out in all parturients with progressive and strong headache after failure of an epidural blood patch, and in some patients even before its placement. Early diagnosis and appropriate treatment are essential for a successful recovery because severe morbidity as well as mortality can occur following unrecognized CVT and SDHs, especially if these intracranial complications appear simultaneously, as it was in our case.
